# Immediate effects of photobiomodulation with low-level laser followed by the voiced tongue trill technique in women with behavioral dysphonia

**DOI:** 10.1590/2317-1782/e20250171en

**Published:** 2026-07-03

**Authors:** Viviane Souza Bicalho Bacelete, Andréa Rodrigues Motta, Elisa Meiti Ribeiro Lin Plec, Ana Cristina Côrtes Gama

**Affiliations:** 1 Programa de Pós-graduação em Ciências Fonoaudiológicas, Faculdade de Medicina, Universidade Federal de Minas Gerais – UFMG - Belo Horizonte (MG), Brasil.; 2 Departamento de Fonoaudiologia, Faculdade de Medicina, Universidade Federal de Minas Gerais – UFMG - Belo Horizonte (MG), Brasil.

**Keywords:** Dysphonia, Larynx, Low-Level Light Therapy, Voice, Rehabilitation, Speech, Language and Hearing Sciences

## Abstract

**Purpose:**

To evaluate the immediate effects of low-power laser photobiomodulation followed by the voiced tongue trill technique (VTTT) in women with behavioral dysphonia.

**Methods:**

This is an experimental study with 30 dysphonic women divided into two groups: Group 1 (G1): placebo LASER followed by VTTT; and Group 2 (G2): infrared LASER at 9 Joules (J) per point (total 63 J) followed by VTTT. Multidimensional assessment included auditory-perceptual evaluation, aerodynamic measurement of maximum phonation time (MPT), acoustic analysis with short-term measurements, cepstral measurements, and extraction of multiparametric indexes, evaluation of self-perceived phonatory effort, and visual-perceptual evaluation of laryngeal images.

**Results:**

Vocal quality did not differ significantly between the intervention moments or between groups in sustained vowel, but there were positive changes in the overall degree of vocal deviation in the continuous speech task in the placebo LASER group followed by VTTT (p=0.047). G1 had a decrease in jitter (0.025) and pitch perturbation quotient (0.033), and G2 had an increase in MPT (p-value=0.013) and vowel CPPS (p-value=0.027), a decrease in self-perceived phonatory effort (p-value=0.014), and an improvement in vocal fold vibration parameters (p-value=0.017).

**Conclusion:**

The results show that infrared LASER associated with VTTT improves MPT, voice harmonic structure, self-perceived phonatory effort, and vocal fold vibration pattern of dysphonic women, suggesting that this resource has potential effects on the voice.

## INTRODUCTION

Dysphonia is any deviation that may impact natural voice production and affect respiratory, glottal, resonant, and/or articulatory levels^(1)^. Vocal disorders occur in 2% to 17% of the general population, with an estimated incidence of up to 44% among professionals with high vocal demands, affecting almost a third of the population at some point in their lives^(2)^.

Although the diagnostic classification and terminology of dysphonia remain problematic, the most current proposal suggests a behavioral/functional and organic dichotomy. Organic dysphonia stems from causes unrelated to vocal use, while behavioral dysphonia encompasses changes whose etiopathogenesis is complex and multifactorial, including anatomical and functional predispositions, inappropriate vocal use, muscle tension, and psycho-emotional factors^(3)^.

Behavioral dysphonia has wide clinical and laryngeal variability. Vocal rehabilitation is the treatment of choice, according to the clinical needs identified in the multidimensional assessment of the voice. Therapeutic approaches aim to balance the respiratory, phonatory, and resonant subsystems and increase the strength, endurance, and flexibility of the laryngeal muscles^(4)^.

The voiced tongue trill technique (VTTT) and semi-occluded vocal tract exercises (SOVTE) stand out among the vocal exercises, with wide clinical application. Their benefits include smoothed glottal contact, pressure balance, improved mucosal wave movement, and greater vocal endurance^(5)^. These techniques enhance source-filter interaction and reduce vibration impact, activating oral, laryngeal, and thoracic muscle groups with high metabolic demand^(5)^_._

Photobiomodulation therapy (PBMT) has emerged as an adjunct strategy for rehabilitation. This is a form of non-invasive light therapy using non-ionizing sources that act on cellular energy metabolism, increasing mitochondrial membrane potential and the enzymatic activity of the electron transport chain, resulting in metabolic changes, increased sodium and potassium (Na-/K+) pump efficiency, and cellular energy synthesis^(6)^. Therapeutic light has an ergogenic effect by accelerating metabolic and structural changes in the muscle, promoting performance, recovery, and resistance to fatigue^(7)^.

The clinical reasoning behind recommending PBMT in voice therapy is guided by knowledge from related areas, based on the premise of metabolic and structural changes in the muscle, which, besides improving performance and accelerating muscle recovery, reduces fatigue and muscle damage^(7)^. There is evidence supporting the anti-edematous, anti-inflammatory, and healing effects of irradiation, which may benefit vocal clinic rehabilitation, since the vast majority of phonotraumatic injuries involve inflammatory and edematous processes in the vocal folds (VF)^(7,8)^.

Although voice clinics use PBMT for its potential modulation of VF inflammation and healing, with a possible analgesic effect and biomechanical improvement via cellular bioenergetics, the evidence is still limited^(8)^. Some in vitro and in vivo studies suggest modulation of inflammatory and healing processes in laryngeal tissues^(8)^.

Given the lack of sufficient evidence available on the immediate effect of PBMT on vocal clinics, the use of low-power LASER requires a number of questions to be clarified for clinical guidance.

Studies evaluating the effect of intervention on the clinical status of subjects with a specific clinical condition (phase 2 experimental studies)^(9)^ are important to promote future studies with higher levels of evidence. Hence, this study’s research question supporting the experiment was, "Does the application of infrared low-level LASER followed by VTTT produce more positive effects on auditory-perceptual, acoustic, laryngeal, and self-perception responses than the application of a placebo followed by the same vocal exercise protocol in women with behavioral dysphonia?”. This research is justified by the need to elucidate the immediate effects of low-level LASER applied before exercise protocol in women with behavioral dysphonia. Given that the translational research movement aims to bring scientific knowledge closer to clinical routine, the analysis of the immediate effect of PBMT on the voice will allow us to better target clinical practice, identify best procedures, and support future research designs with higher levels of evidence.

## METHODS

This randomized controlled experimental study was approved by the Research Ethics Committee of the Federal University of Minas Gerais (UFMG), Brazil, under protocol number 4.704.038. The study was registered as a clinical trial on the Brazilian Clinical Trials Registry (ReBEC) platform and followed the recommendations of the Consolidated Standards of Reporting Trials (CONSORT) for clinical trials^(10)^.

The energy of 9 J was chosen for the experiment based on the results of a previous study in a vocally healthy population^(11)^. The study was conducted at the Speech-Language Functional Health Observatory of UFMG’s Medical School. The volunteers agreed to participate in the data collection by signing an informed consent form. They were recruited through invitations to professionals with high vocal demands in the community and dissemination of the research on social networks, constituting a convenience sample.

The inclusion criteria were women aged 18 to 55 years, with behavioral dysphonia, abnormal laryngeal examination, body mass index (BMI) below 30^(12)^, and the ability to comfortably perform VTTT.

This study considered behavioral dysphonia as the presence of vocal complaints and symptoms, and/or abnormal vocal quality in the auditory-perceptual evaluation, and changes in the laryngeal examination characterized by phase asymmetry, benign VF lesions, and glottal chink (except for the posterior glottal chink, considered physiological)^(13)^.

Participants who reported a negative self-perception of voice quality, answering positively to the question, “Do you currently consider yourself to have a voice problem?”, were invited to undergo a screening process, which included a voice evaluation by a speech-language pathologist and, if necessary, an otolaryngological assessment. They initially administered the Voice Symptom Scale (VoiSS); total scores equal to or greater than 16 points were considered indicative of vocal impairment^(14)^.

Then, a researcher with over 10 years of experience conducted an auditory-perceptual evaluation of voice and analyzed the overall degree of vocal deviation (G) using a 4-point scale: neutral (no perceptible auditory deviations), mild, moderate, and severe. This evaluation was based on two vocal tasks: sustained vowel /a/ and continuous speech (days of the week). Also, another experienced speech-language pathologist analyzed the voice samples independently. In cases where both evaluators confirmed any degree of change in the overall degree of vocal deviation (from mild to severe) or presence of vocal complaints and symptoms, participants were referred to an otolaryngologist for laryngeal examination.

The laryngeal assessment was performed by a single otolaryngologist using high-speed video laryngoscopy (VAV). Benign VF lesions, phase asymmetry, and incomplete glottal closure (except for physiological glottal chink) were considered abnormal^(13)^. Laryngeal diagnosis confirmation required agreement between two independent otolaryngologist raters.

Participants were diagnosed with functional (behavioral) dysphonia based on the combination of a negative self-perception of voice quality, an abnormal VoiSS score, and/or overall degree of vocal deviation (G), and laryngeal changes consistent with benign lesions, according to the multidimensional voice assessment criteria.

The exclusion criteria were pregnant, menstrual, and premenstrual women; those with an allergic reaction and/or respiratory crises on the day of the evaluation; endocrine, neurological, or neoplastic diseases; smokers; those who had already undergone laryngeal surgery; those who had photosensitivity or some skin disease/injury; tattoos in the region where the light was applied; those who reported taking medications for skin treatments or who were unable to perform the VTTT.

Having met the eligibility criteria, 30 women were selected and allocated through stratified randomization by laryngeal diagnosis, equally divided into the groups. Each block was randomly assigned by a simple draw performed by the researcher to allocate the groups. The participants were blinded to the procedure.

After randomization and allocation, all participants were distributed equally into two groups (15 participants in each):

- **Group 1 (G1 - Placebo LASER + VTTT)** – Placebo LASER (device emitting light but not energy) for 90 seconds in seven anatomical points of the larynx^(15)^; immediately afterwards, they performed VTTT for 5 minutes^(16)^.
- **Group 2 (G2 - Experimental LASER + VTTT) –** infrared LASER at 9 J per point for 90 seconds at three points in each hemilarynx and one central point, with a total 63 J dose in the larynx^(11,15)^. Immediately after applying the LASER, they performed the VTTT for 5 minutes^(16)^.

**Figure 1:**
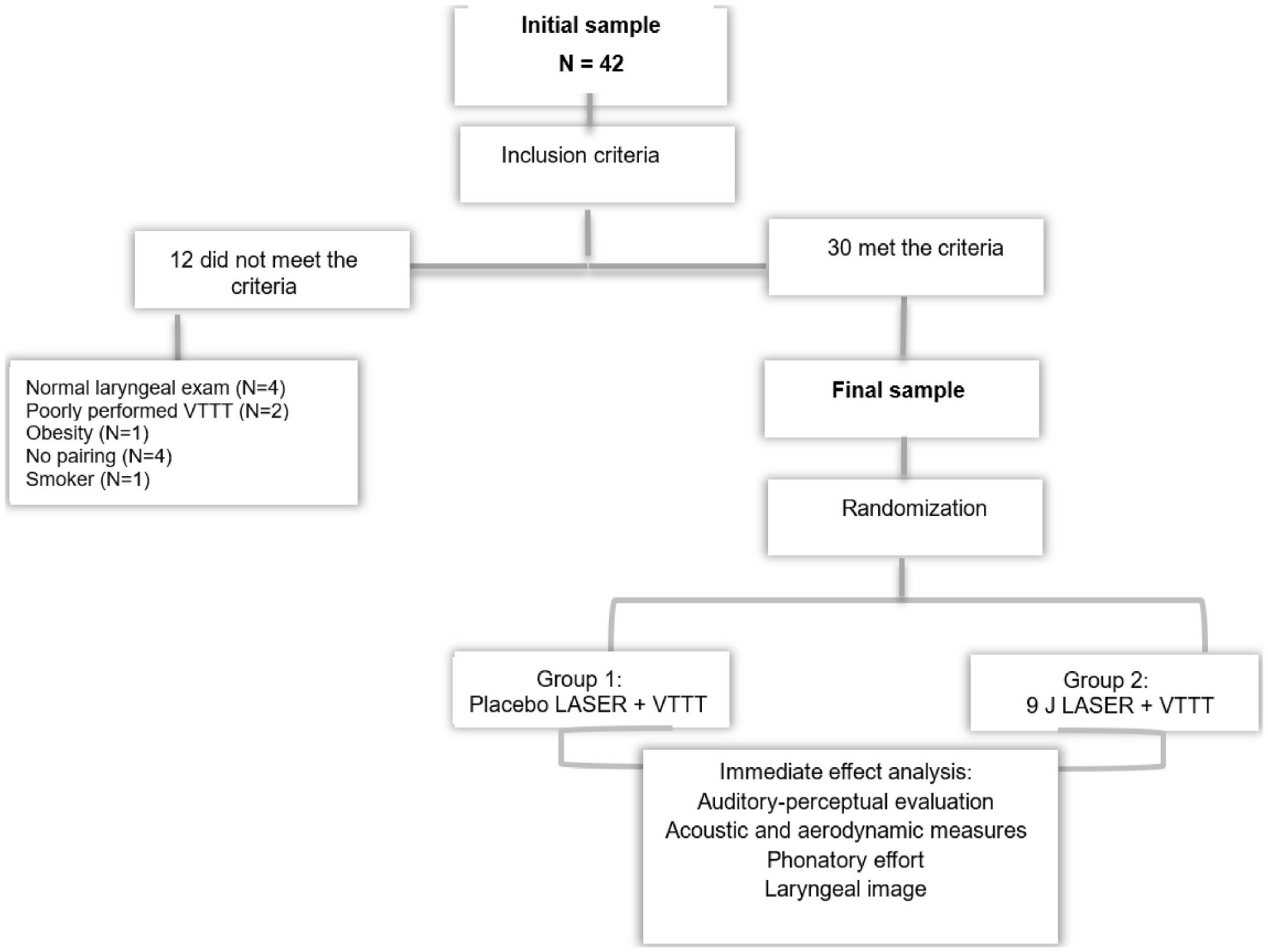
Sample selection

The sample selection process and study group distribution are shown in Figure 1.

**Caption:** J = joules; N = number; VTTT = voiced tongue trill technique

### Sample characteristics

In G1, the mean age was 37 years (minimum = 22; maximum = 49; SD: 8.17), and the BMI was 25.3 (minimum = 20.5; maximum = 29; SD: 2.18). Regarding the skin phototype (Fitzpatrick Scale) (26), six (40%) participants had white skin (Types I and II), three (20%) had light brown skin (Type III), two (13.3%) had moderate brown skin (Type IV), three (30%) had dark brown skin (Type V), and one (6.7%) had black skin (Type VI). The laryngeal diagnosis found one (6.7%) participant with phase asymmetry, one (6.7%) with VF nodules, two (13.3%) with VF thickening, two (13.3%) with an anterior chink, four (26.7%) with double chink, and five (33.3%) with mid-posterior double chink.

In G2, the mean age was 34 years (minimum = 19; maximum = 46; SD: 8.68), and the BMI was 24.27 (minimum = 20; maximum = 29; SD: 3.16). Regarding skin phototype^(17)^, five (33.3%) participants had white skin (Types I and II), five (33.3%) light brown skin (Type III), two (13.3%) moderate brown skin (Type IV), one (6.7%) dark brown skin (Type V), and two (13.3%) black skin (Type VI). Laryngeal diagnoses were precisely the same as in G1.

### Baseline measures

In G1, the number of tongue trills ranged from 20 to 40 repetitions (mean = 30; SD: 6.16), and the VoiSS score ranged from 16 to 64 (mean = 39; SD: 13.05), with the greatest impact on the impairment aspect (23%) (mean = 23; minimum = 7; maximum = 46; SD: 10.9).

In G2, the number of tongue trills ranged from 17 to 43 repetitions (mean = 31; SD: 8), and the VoiSS score ranged from 16 to 87 (mean = 39; SD: 20.21), also with a greater impact on impairment (21.8%) (mean = 22; minimum = 4; maximum = 43; SD: 10.3).

The groups were matched regarding age (p = 0.442), BMI (p = 0.350), skin phototype (p = 0.750), laryngeal diagnosis (p = 0.999), VoiSS (p = 0.884), and number of tongue trills (p = 0.662).

### PBMT

A DMC device, model Therapy EC, was used to apply the LASER, with a power of 100 mW and an output spot of ​0.0984 cm^2^ in the infrared wavelength (808 ± 10 nanometers) at 9 J per point, in seven points in the larynx, as a placebo or before the VTTT. The dosimetric parameters are described in detail in Table 1.

**Table 1:** Photobiomodulation parameters with low-level LASER

**Dosimetric parameters**	**Placebo LASER + VTTT**	**Experimental LASER + VTTT**
Wavelength (nm)	NA	808 ± 10
Equipment power (mW)	NA	100
Output spot (cm^2^)	NA	0.0984
Power density of power or irradiance (W/cm^2^)	NA	1.01
Energy density or flow (J/cm^2^)	NA	91.5
Emission mode	NA	continuous
Irradiation application mode	NA	per point
Energy per point (J)	NA	9
Total points irradiated	NA	7
Irradiation time per point (s)	NA	90
Total energy (J)	NA	63

**Caption:** cm^2^ = square centimeters; J = joule; J/cm^2^ = joule per square centimeter; mW = milliwatts; NA = not applicable; nm = nanometers; s = seconds; W/cm^2^ = watt per square centimeter

**Figure 2:**
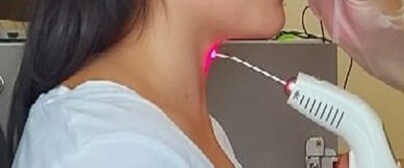
Application of low-level LASER in the larynx

**Source:** Plec et al.^(15)^

LASER was applied with the participants sitting on a chair, and both the applicator and the participant wore personal protective equipment for the eyes. Before irradiating the therapeutic light, the skin was cleaned by rubbing the neck with 70% alcohol. The tip of the equipment was covered with PVC plastic film, and the application on the neck was continuous and per point (Figure 2).

**Figure 3:**
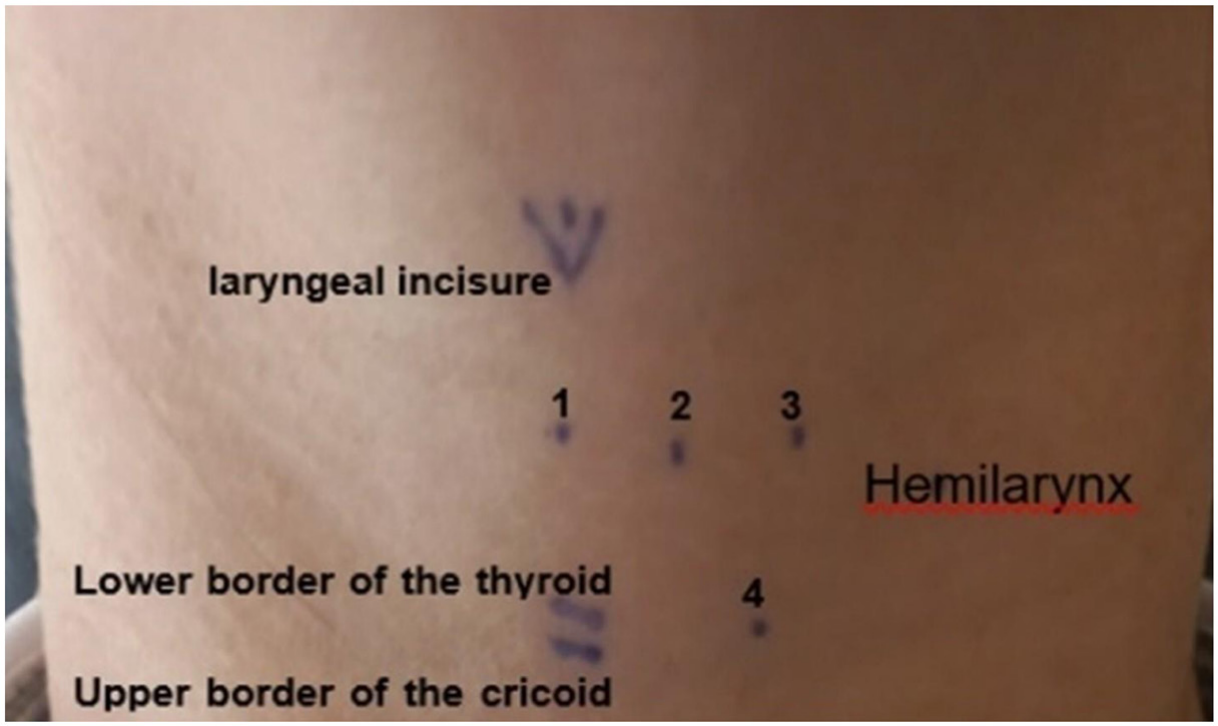
Schematic representation of the LASER application points in the larynx

The anatomical limits to locate the glottic level and the main intrinsic laryngeal muscles were demarcated according to previous research^(15)^. One central point and three in each hemilarynx were identified, totaling seven points, to apply light in the neck^(15)^. Point 1 was defined in the topography of the anterior commissure of the larynx; point 2, in the region of the thyroarytenoid muscle and mucous membrane; point 3, aimed to reach the lateral cricoarytenoid muscle; and point 4, in the topography of the cricothyroid muscle. A schematic representation of the anatomical points is shown in Figure 3.

**Caption:** 1- Point 1 (anterior commissure of the larynx); 2- Point 2 (membranous portion of the vocal fold – thyroarytenoid muscle and mucous membrane); 3- Point 3 (cartilaginous portion of the vocal fold - lateral cricoarytenoid muscle); 4- Point 4 (cricothyroid muscle). **Source:** Plec et al.^(15)^

The researcher applying the treatment followed the same procedures for both groups and manipulated the equipment buttons for it to sound a beep without emitting therapeutic light in G2.

### Voiced Tongue Trill Technique

The PBMT was applied experimentally or as a placebo, followed by the VTTT performed standing with the prolonged phoneme /r/^(16)^. The VTTT was previously demonstrated and explained to each participant, with emphasis on performing it at habitual frequency and intensity, without inducing laryngeal, articulatory, or respiratory effort. Participants were instructed to keep their lips in a neutral, relaxed position, slightly open, avoiding facial tension that could interfere with performing the technique. The vocalization was to be continuous, stable, and comfortably sustained, with spontaneous breathing pauses whenever necessary, according to each one's physiological limits. The task consisted of performing the technique for 5 minutes, seated, and supervised by the lead researcher, who monitored the rhythm and breathing, timed the exercise using a digital stopwatch, and recorded the number of task repetitions per period.

### Evaluation of outcome variables

**Figure 4:**
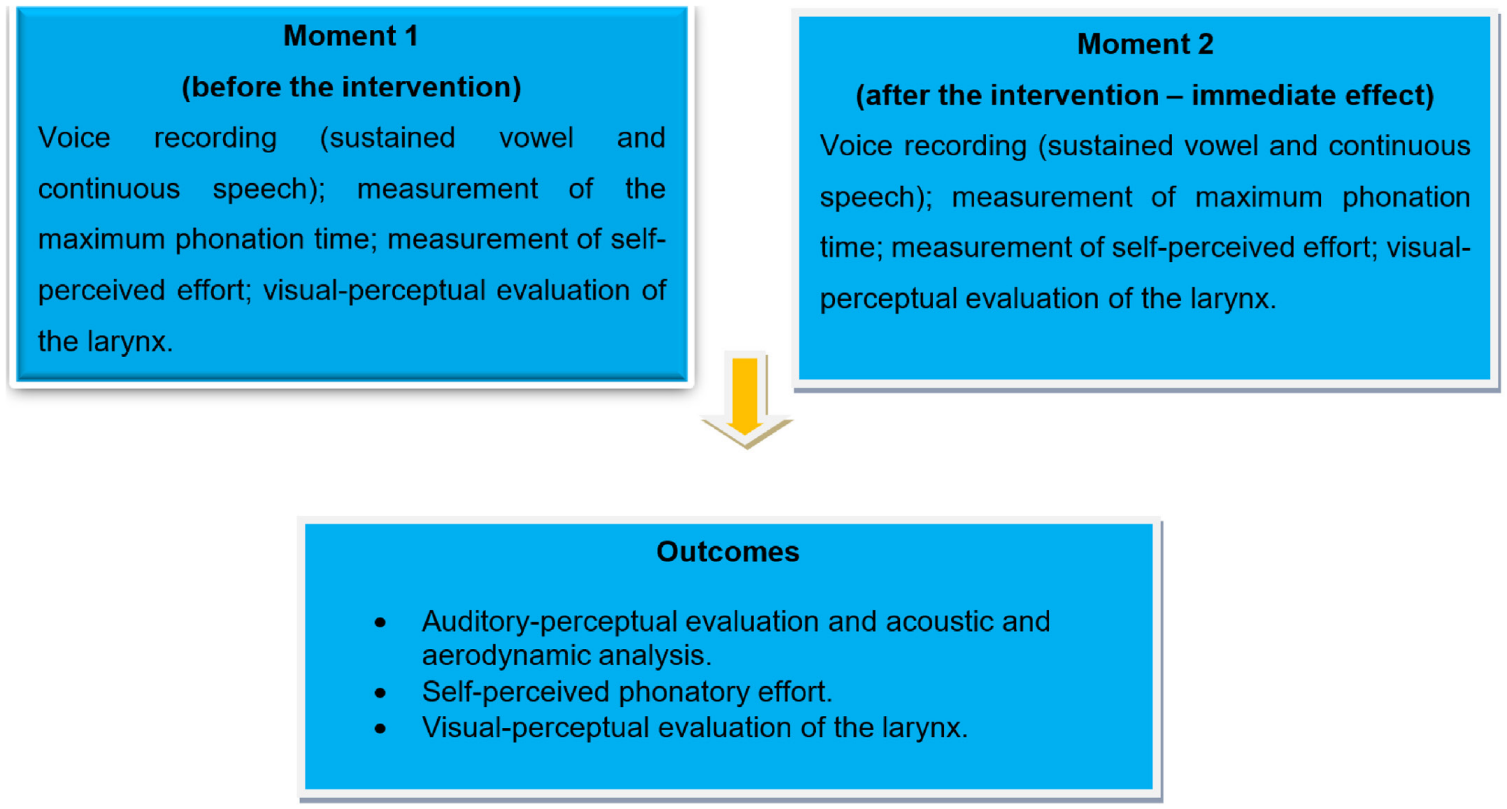
Study stages

The following dependent variables were used to analyze the immediate effect of low-level LASER: 1) auditory-perceptual evaluation of vocal quality; 2) acoustic analysis of voice and aerodynamic measurement; 3) self-perceived phonatory effort; and 4) visual-perceptual evaluation of laryngeal images. The outcome variables were evaluated in the groups at two moments: before (moment 1) and immediately after the intervention (moment 2). The procedures were performed in a single meeting (Figure 4).

All procedures for collecting dependent variables are detailed below.

### Voice recording and auditory-perceptual evaluation

Voice samples were captured in an acoustically treated room by a Samson C01 condenser microphone, positioned at 45 degrees and 10 centimeters from the corner of the mouth, and recorded directly into a computer system (Dell Optiplex GX260 computer with a DirectSound^®^ professional sound card). Volunteers remained standing during recording, emitting a sustained vowel /a/, and counting from 1 to 20 at their usual pitch and loudness.

Voice quality was analyzed by four speech-language pathologists with more than 10 years of experience in auditory-perceptual evaluation, blinded to the intervention and assessment moment. The sustained vowel and continuous speech samples were randomized, not revealing the intervention moment.

The judges evaluated the degree of vocal deviation (overall impression of vocal quality) in the sustained vowel and continuous speech samples separately. After listening to each voice in the separate blocks, they quantified the overall degree of vocal deviation (G) by marking a 100-millimeter visual analog scale (VAS), in which 0 indicated no vocal deviation, and the right end (100), intense deviation ^(18)^.

Also, 20% of the 60 sustained vowels and 60 continuous speech samples were randomly selected for reanalysis (12 sustained vowels and 12 continuous speech samples per rater, totaling 72 samples per type) to verify intrarater agreement. The intraclass correlation coefficient (ICC) was used in the PAST^©^ program^(19)^, and the raters had an agreement of 76%, 80%, 92%, and 93%^(19)^. The responses of the three raters with good to excellent agreement were considered for analysis, using their mean response values.

### Acoustic measure analysis

Voices were recorded with the Kay Pentax^®^ Computerized Speech Lab (CSL) program, model 6103, Multi-Dimensional Voice Program (MDVP) module^(20)^. The following parameters were used to extract aerodynamic and acoustic measures:

1. Maximum phonation time (MPT): Measured from the sustained vowel (/a/) task three times, considering the mean of the emissions. The normal range is 15 to 25 seconds^(1)^.
2. Fundamental frequency (^f^o): Mean of all extracted frequency periods. In women, values may range from 150 to 250 hertz (Hz)^(20)^.
3. Jitter and pitch perturbation quotient (PPQ): A measure of short-term frequency disturbance, with a normal value of up to 0.36%^(20)^.
4. Shimmer and amplitude perturbation quotient (APQ): Measure of short-term amplitude disturbance, with a normal value of up to 3.81%^(20)^.
5. Harmonics-to-noise ratio (HNR): A measure of noise that relates the harmonic component to the noise component of the acoustic wave. Its normal value is up to 0.11 dB^(20)^.
6. Cepstral measures: Cepstral peak prominence (CPP) was measured using the Praat computer program, version 6.2.23, by selecting the best part of the sustained vowel /a/ emission, totaling 3 central seconds, and the entire continuous speech emission^(21)^. The VOXplot program extracted cepstral peak prominence-smoothed (CPPS) when extracting multiparametric index values. The cutoffs were 28.77 dB for CPP in sustained vowel, 17.02 dB for CPPS in sustained vowel, 28.58 dB for CPP in speech, and 11.3 dB for CPPS in speech^(21)^.
7. Multiparametric acoustic indices: The samples were edited in the VOXplot software and consisted of 3-second averages of the sustained vowel /a/ at usual frequency and intensity, and counting from 1 to 10. The values ​​of the Acoustic Breathiness Index (ABI) and Acoustic Voice Quality Index (AVQI) were obtained through a script in the Praat software. The AVQI and ABI have already been validated for Brazilian Portuguese, with established cutoffs of 1.33 and 2.94, respectively^(22,23)^.

### Analysis of self-perceived phonatory effort

The self-perceived phonatory effort was analyzed using the Borg CR10-BR Scale adapted for vocal effort, in which 0 corresponded to no vocal effort, and 10 corresponded to maximum effort^(24)^.

The sustained vowel emission task was used as a reference for analyzing self-perceived phonatory effort. Participants were instructed as follows before applying the scale: "You will evaluate how much effort you made when using your voice during the task. Use this scale from 0 to 10, where 0 means no vocal effort, and 10 means the greatest effort you could make to speak. Choose the number that best represents the effort you made while speaking."

After the sustained vowel emission, the numeric scale was visually presented to the participant, with the endpoints highlighted ("0 = no effort" and "10 = maximum effort"), so that the participant could immediately indicate the number corresponding to the perceived effort during the task.

### High-speed video laryngoscopy

Laryngeal images were obtained with high-speed video laryngoscopy, recorded at 2000 frames per second, using a 70° rigid laryngoscope with 300 W xenon light (KayPentax^®^, Lincoln Park, New Jersey^®^), model 9710, resolution of 512 x 512 pixels with 8-bit RGB color mode.

The participants remained seated with a slight anterior cervical projection and received topical anesthesia with 10% lidocaine in the oral cavity before the examination. They were instructed to emit the vowels /i/ and /ε/ at their usual frequency and intensity.

A single physician performed the examinations, and four otorhinolaryngologists with more than 10 years of experience in laryngeal evaluation were invited to classify the high-speed video laryngoscopy videos. The pairs of examinations designated A or B were randomized, not revealing the intervention moment, and the analyses were performed in pairs by comparison. Also, 20% of the sample was replicated to analyze intrarater agreement, using Kappa statistics^(25)^. The answers of the three that had moderate (65% and 67%) and almost perfect (100%) agreement were chosen, using the mode value of the judges’ answers^(25)^.

The following categorization was used to tabulate the responses:

- If the laryngeal image was considered better after the experiment = improved.
- If the laryngeal image was considered better before the experiment = worsened.
- If the laryngeal images were considered the same = equal.

The visual-perceptual evaluation of the images was performed using an adapted version of the vocal fold vibration pattern assessment for VAV, whose evaluated parameters were: glottic closure (appearance of the glottis during the most closed portion of the glottic cycle), mucosal wave (magnitude of mucosal wave motion), amplitude (magnitude of lateral movement of the vocal folds), phase symmetry (degree to which the vocal folds move as mirror images of each other), and glottic cycle periodicity (consistency of glottic cycles)^(26)^.

### Statistical analysis

The study used R-STUDIO (version 4.1.0) and SPSS statistical software (version 25). The Wilcoxon statistical test was used for intra-group non-parametric analyses before and after the experiment, and the Mann-Whitney test was used for comparisons between groups. The paired t-test was used for parametric analyses, and the chi-square test was used for statistics on categorical variables. A 5% significance level was used in all analyses.

## RESULTS

Table 2 shows the results of the auditory-perceptual evaluation. In the sustained vowel task, the mean vocal deviation values were similar between the groups before and after the intervention, with no statistically significant difference. Continuous speech improved significantly in the LASER placebo group associated with VTTT (p = 0.047). No significant differences were observed between the groups after the interventions.

**Table 2:** Results of the auditory-perceptual assessment of the overall degree of vocal deviation using the visual analog scale in the experimental and placebo LASER groups associated with VTTT

		**G2 - Experimental LASER+VTTT**					**G1 - Placebo LASER+VTTT**						**Experimental vs. Placebo**
		**mean**	**median**	**SD**	**min**	**max**	**mean**	**median**		**SD**	**min**	**max**	**p-value** **b**
Vowel	Before	37.67	34.00	16.40	22.00	81.00	28.07	29.00	8.24		14.00	42.00	0.064
	After	34.53	30.00	17.49	15.00	84.00	26.53	26.00	7.58		16.00	45.00	0.135
	*P-value* *a*	0.064					0.432						
Speech	Before	32.07	23.00	23.05	5.00	82.00	21.27	16.00	18.08		4.00	59.00	0.115
	After	31.07	21.00	24.79	8.00	90.00	18.67	8.00	18.45		3.00	60.00	0.058
	*P-value^a^*	0.706					**0.047**						

a Wilcoxon Signed Ranks Test for intra-group comparison;

b Mann-Whitney test for comparison between experimental and placebo groups

Statistically significant values are presented in bold (p < 0.05);

**Caption:** SD = standard deviation; max = maximum; min = minimum; VTTT = voiced tongue trill technique

Table 3 presents aerodynamic and acoustic measurement values.

**Table 3:** Comparative analysis of maximum phonation time, short-term acoustic measurements, cepstral measurements, and multiparametric acoustic indexes between experimental and placebo LASER groups associated with VTTT

		**Experimental LASER + VTTT**					**Placebo LASER + VTTT**					**Experimental vs. Placebo**
		**Mean**	**Median**	**SD**	**Min**	**Max**	**Mean**	**Median**	**SD**	**Min**	**Max**	**p-value** **b**
MPT	Before	9.13	9.00	2.90	4.00	14.00	10.60	10.00	2.10	7.00	14.00	0.177
	After	10.13	10.00	3.89	4.00	18.00	10.80	10.00	2.83	7.00	16.00	0.644
	*P-value* *a*	**0.013**					0.590					
fo	Before	205.39	211.70	32.11	130.21	248.82	199.60	203.80	30.85	142.20	255.96	0.373
	After	201.95	206.76	26.78	133.68	242.06	206.93	210.89	33.92	145.13	265.43	0.633
	*P-value* *	0.510					0.100					
JITTER	Before	1.68	1.45	1.00	0.58	4.16	1.57	1.29	0.69	0.59	2.88	0.983
	After	1.71	1.40	1.16	0.60	5.11	1.19	0.94	0.80	0.29	2.91	0.158
	*P-value^a^*	0.865					**0.025**					
SHIMMER	Before	4.14	3.91	1.65	2.41	8.33	3.60	3.29	1.61	0.41	6.83	0.455
	After	3.93	3.14	2.32	1.21	9.43	3.21	3.00	0.87	1.84	5.06	0.678
	P-value*	0.766					0.233					
PPQ	Before	1.00	0.83	0.63	0.36	2.52	0.92	0.74	0.40	0.36	1.66	0.934
	After	1.01	0.80	0.73	0.35	3.21	0.69	0.53	0.46	0.17	1.64	0.184
	*P-value* *	0.851					**0.033**					
APQ	Before	2.97	2.70	1.25	1.68	5.80	2.78	2.61	0.91	1.64	4.73	0.772
	After	3.37	2.24	2.15	1.32	8.89	2.32	2.13	0.68	1.25	4.00	0.372
	P-value*	0.496					0.135					
HNR	Before	0.13	0.13	0.02	0.09	0.17	0.12	0.12	0.02	0.07	0.16	0.294
	After	0.13	0.11	0.04	0.08	0.24	0.13	0.13	0.02	0.09	0.16	0.400
	*P-value^a^*	0.861					0.596					
CPP_VOWEL	Before	22.79	23.15	3.19	15.65	28.35	24.16	24.35	1.80	21.00	27.00	0.141
	After	23.21	24.05	2.63	15.55	26.20	24.99	24.75	2.66	21.00	30.00	0.158
	*P-value^a^*	0.268					0.080					
CPPS_VOWEL	Before	12.66	14.00	3.57	6.00	17.00	15.01	15.30	1.65	11.70	18.40	**0.049**
	After	14.11	14.45	2.11	7.95	16.65	14.98	14.50	2.16	12.15	19.50	0.604
	*P-value^a^*	**0.027**					0.938					
CPP_SPEECH	Before	16.80	16.40	1.41	14.90	19.55	17.11	17.00	1.02	15.15	18.70	0.330
	After	16.88	16.50	1.17	14.80	19.00	17.12	17.60	1.19	15.10	19.25	0.506
	P-value*	0.635					0.965					
CPPS_SPEECH	Before	11.78	11.45	2.28	8.72	16.30	12.52	12.60	1.46	10.25	15.20	0.290
	After	12.00	12.18	1.79	8.34	15.00	12.22	12.30	1.79	9.30	15.60	0.709
	P-value*	0.591					0.306					
AVQI	Before	3.62	3.30	2.27	0.22	7.19	2.27	2.10	1.01	0.35	4.05	0.120
	After	2.81	2.80	1.51	0.50	6.74	2.37	2.10	1.17	0.13	4.05	0.561
	P-value*	0.075					0.531					
ABI	Before	4.57	4.00	2.04	2.10	8.33	3.46	3.30	0.94	1.45	4.80	0.171
	After	3.96	3.70	1.28	2.20	7.61	3.44	3.30	1.02	1.20	5.00	0.350
	P-value*	0.088					0.857					

a Wilcoxon Signed Ranks Test for intra-group comparison;

b Mann-Whitney test for comparison between experimental and placebo groups;

* Paired t-test;

Statistically significant values are presented in bold (p < 0.05);

**Caption:** SD = standard deviation; max = maximum; min = minimum; MPT = maximum phonation time; APQ = amplitude perturbation quotient; CPP = cepstral peak prominence; CPPS = cepstral peak prominence-smoothed; dB = decibel; **fo** = fundamental frequency; HNR = harmonics-to-noise ratio; PPQ = pitch perturbation quotient; s = seconds; ABI = Acoustic Breathiness Index; AVQI = Acoustic Voice Quality Index

G2’s mean MPT and the CPPS vowel measures increased significantly post-intervention (p = 0.013 and 0.027). In G1, the mean jitter and PPQ values decreased significantly post-intervention (p = 0.025 and 0.033, respectively). The mean CPPS vowel measures differed significantly between the groups before the intervention (p = 0.049). No significant differences were observed between the groups after the interventions.

Table 4 shows the mean values of self-perceived phonatory effort before and after the intervention for both groups. The mean value of self-perceived phonatory effort was significantly lower in G2 post-intervention (p-value = 0.014). No significant differences were observed between the groups after the interventions.

**Table 4:** Analysis of self-perceived phonatory effort in the experimental and placebo LASER groups associated with VTTT

		**Experimental LASER + VTTT**					**Placebo LASER + VTTT**					**Experimental vs. Placebo**
		**mean**	**median**	**SD**	**min**	**max**	**mean**	**median**	**SD**	**min**	**max**	**p-value** **b**
Phonatory effort	Before	3.40	1.00	3.79	0.00	10.00	3.07	0.00	3.86	0.00	10.00	0.824
	After	1.53	0.00	2.36	0.00	8.00	2.47	0.00	3.48	0.00	10.00	0.639
	*P-value* *a*	**0.014**					0.100					

a Wilcoxon Signed Ranks Test for intra-group comparison;

b Mann-Whitney test for comparison between experimental and placebo groups;

Statistically significant values are presented in bold (p < 0.05);

**Caption:** SD = standard deviation; max = maximum; min = minimum

The results in Table 5 show that the group with experimental LASER associated with VTTT improved laryngeal parameters (glottal closure, phase symmetry, mucosal wave movement, and glottic cycle periodicity) more often, with a significant difference between the groups (p = 0.017).

**Table 5:** Comparative analysis between experimental and placebo LASER groups associated with VTTT in the visual-perceptual evaluation of the larynx

		**Experimental**	**Placebo**	**Total**	**P-value**
VISUAL-PERCEPTUAL EVALUATION OF THE LARYNX	Worsened	4 (26.7%)	5 (33.3%)	9 (30%)	**0.017**
	Equal	2 (13.3%)	8 (53.3%)	10 (33.3%)	
	Improved	9 (60%)	2 (13.3%)	11 (36.67%)	

Pearson chi-square test;

Statistically significant values are presented in bold (p < 0.05)

## DISCUSSION

Although this study did not statistically compare the sustained vowel and continuous speech tasks, the descriptive results suggest greater vocal deviation during the sustained vowel emission. This trend is consistent with findings in the literature, which indicate that sustained vowel tasks tend to reveal deviations related to the glottal source more prominently, resulting in higher perceptual ratings of dysphonia^(27)^.

The literature indicates that VTTT may produce immediate effects on the glottal source, such as the reduction of breathiness and roughness in dysphonic women^(16)^. In the present study, neither group had statistically significant differences in the sustained vowel task before and after the intervention, although numerical reductions in vocal deviation scores were observed. Based on these data, it is not possible to infer positive effects in either group solely from descriptive analysis. These results may have been influenced by the small sample size, which possibly limited the statistical power to detect small or moderate differences between interventions in this task.

A positive effect on vocal quality during linked speech was observed in the group that received a placebo LASER associated with VTTT. This agrees with other studies that demonstrated improvements in vocal projection, intensity, and resonant balance following the application of VTTT^(16)^. This improvement may be attributed to the effects of the exercise, in which the vocal tract actively participates in energy production, and the source–filter interaction enhances vocal intensity, efficiency, and energy conservation^(16)^. Although a reduction in auditory-perceptual scores during the continuous speech task was observed in the group that received experimental LASER combined with VTTT, this difference did not reach statistical significance. This result may be related to the reduced sample size or individual variability in participants' responses. The limited number of subjects may have compromised the statistical power of the analysis, making it difficult to detect subtle differences between groups, especially with a small effect size.

Only the group that received experimental LASER associated with VTTT had a significant increase in aerodynamic MPT measures. The latter is frequently used as a comparison parameter in various interventions, as a marker of therapeutic efficacy by reflecting improvements in glottal closure and myoelastic and aerodynamic control^(28)^. These findings suggest that the observed increase in the experimental group may be related to the applied intervention, but they also indicate that the study may not have had sufficient power to detect differences between groups, particularly if the effect size is small or moderate. Therefore, despite the promising findings within the experimental group, future studies with larger samples are needed to confirm these results and to determine whether the differences between groups remain significant with greater statistical power.

The decrease in jitter and PPQ in the group with placebo LASER associated with VTTT can be justified by the improved periodicity of VF vibration through muscular and functional laryngeal adjustments and vibration pattern changes after exercise^(1)^. The lack of statistical significance in the experimental group associated with VTTT may be related to the sample size.

The vowel CPPS measure increased in the group with experimental LASER associated with VTTT, representing a better-defined harmonic structure possibly related to improved mucosal wave movement^(21)^. Regarding the multiparametric indices, ABI and AVQI scores decreased, particularly in the experimental group, suggesting reduced breathiness and roughness after the intervention, although without statistical significance. This lack of significance may be related to the small sample size, which may have limited the statistical power of the analysis and hindered the detection of subtle differences between groups. Cepstral measures are strong predictors of vocal deviation and more robust than traditional acoustic measures^(29)^.

The results of the self-assessed phonatory effort showed an improvement in the perception of effort after applying LASER in the experimental group associated with VTTT. Vocal self-assessment has been highly valued, useful to assess the impact of deviation on the patient's life, monitor the evolution, and evaluate the treatment effectiveness^(30)^. The Borg CR10-BR Scale, adapted for vocal effort, is a specific instrument for self-assessment of vocal effort after a task^(24)^.

The laryngeal examination results revealed an improvement in the VF vibration patterns in the group with experimental LASER associated with VTTT in nine participants (60%), while in the placebo laser group, there was improvement in two cases (13,3%), suggesting immediate positive effects on glottal closure, mucosal wave movement, phase symmetry, and cycle periodicity. High-speed video laryngoscopy allows more precise analysis of the glottic area and mucosal wave movement of the VF^(26)^.

The clinical reasoning that guides PBMT recommendations in ​​voice is driven by knowledge from related areas, based on the premise of metabolic and structural changes in the muscle^(6-8)^. Muscle activation during vocal exercises consumes high energy; hence, applying light to the intrinsic laryngeal muscles before the exercise can enhance therapeutic results^(11)^.

In short, VTTT has positive effects on the quality and acoustic measures of the voice and, when associated with low-level LASER, it increases MPT and vowel CPPS, decreases phonatory effort, and improves the VF vibration patterns. Although some results did not reach statistical significance, the observed effect sizes indicate clinically relevant trends that may have been underestimated due to the reduced sample size. Integrating various types of data is essential for accurate diagnosis and planning/monitoring the effectiveness of vocal therapy^(28-30)^.

This is the first study to evaluate the immediate effects of infrared light on women with vocal disorders. It presents preliminary results regarding the immediate effects of low-level LASER on dysphonic women – hence, we do not yet know the short- and medium-term effects on different clinical voice conditions.

The findings provide valuable parameters to start investigations and better elucidate the effects of low-level LASER on vocal clinical practice when associated with an exercise protocol. Variables that may interfere with tissue penetration, such as sex, age, physiological state of the treated tissue, and skin thickness, were controlled.

Lastly, as this study had a limited sample size, we suggest future research with larger samples, considering other designs. They could use more robust methodological designs, such as randomized controlled clinical trials with blind allocation and stratification by type and degree of dysphonia, as well as crossover models, in which each participant receives both the active intervention and the placebo at different times, with an appropriate washout period. Longitudinal studies are also needed to assess the maintenance of therapeutic effects in the medium and long term. Such designs may enhance the internal and external validity of the results, helping advance knowledge regarding the efficacy of PBMT in the clinical management of functional dysphonia in women.

## CONCLUSION

PBMT with infrared low-level LASER (9 J per point) followed by VTTT produced immediate positive effects in women with behavioral dysphonia. The intervention resulted in immediate improvements in aerodynamic and acoustic parameters, self-perceived phonatory effort, and VF vibration behavior. These findings suggest that low-level LASER may enhance the therapeutic effects of vocal exercises and support its application in clinical voice practice.
